# Epicardial Adipose Tissue Volume and Left Atrial Remodeling: A J-Shaped Association in Older Adults

**DOI:** 10.3390/jcdd13020078

**Published:** 2026-02-04

**Authors:** Xinyue Zhao, Guangjian Wang, Xuefeng Ni, Hui Lian

**Affiliations:** Department of Health Care, Peking Union Medical College Hospital, Chinese Academy of Medical Sciences and Peking Union Medical College, Beijing 100730, China

**Keywords:** epicardial adipose tissue, left atrial enlargement, cardiac remodeling, older population, cross-sectional

## Abstract

Background: Previous studies identified epicardial adipose tissue (EAT) as a metabolic risk factor for atrial remodeling. However, given the distinct physiological changes associated with aging, findings from the general population may not translate directly to older adults. This study aims to clarify the relationship between EAT and left atrial (LA) diameter in older adults specifically. Methods: This retrospective cross-sectional study was conducted among in an older adult cohort (aged ≥ 65 years) at Peking Union Medical College Hospital. The association between EAT and LA diameter was evaluated using multivariable linear regression, a generalized additive model, and restricted cubic spline (RCS) modeling. Results: Among 353 participants (median age 75 years), EAT was independently associated with LA diameter (β = 0.286, *p* < 0.001) after adjusting for confounders including age, BMI, and LDL-C. Notably, RCS analysis revealed a J-shaped relationship between EAT volume and LA dimensions. Specifically, when EAT exceeded 110.7 cm^3^, the LA diameter increased significantly by 0.22 mm per 10 cm^3^ increase in EAT (*p* = 0.004). Conclusions: EAT accumulation shows a non-linear association with left atrial remodeling in older adults, with an identifiable threshold at 110.7 cm^3^. EAT may be a valuable biomarker for cardiovascular risk stratification, suggesting that EAT burden monitoring could be beneficial in older populations.

## 1. Introduction

Epicardial adipose tissue (EAT) is a biologically active visceral adipose tissue located within the pericardial cavity. It is anatomically distinguished from other visceral fat depots by microcirculation shared with the underlying myocardium without a fascial layer [1,2]. This unique anatomical contiguity facilitates a direct cross-talk between adipose tissue and the heart [3]. Under physiological conditions, EAT exhibits metabolic, thermogenic, and mechanical protective characteristics. However, in pathological states, excessive EAT may act as a pro-inflammatory organ, causing “cardio-lipotoxicity”, disrupting equilibrium, and promoting a series of adverse cardiovascular outcomes, such as arrhythmias, pulmonary arterial hypertension, coronary artery disease, and heart failure [1,4,5,6,7,8]. The myocardium is particularly susceptible to paracrine effects from EAT, where secreted inflammatory cytokines and profibrotic mediators, directly contribute to atrial fibrosis, as well as electrical and structural remodeling [9,10].

Left atrial (LA) remodeling is a clinical precursor to atrial fibrillation (AF), stroke, and heart failure. While hemodynamic load and general obesity are traditional determinants of LA remodeling [11], accumulating evidence highlights the specific, independent contribution of EAT. Studies have repeatedly demonstrated a positive correlation between EAT burden and LA size [12,13,14]. A systematic review and meta-analysis quantified this relationship, showing that every 10 mL increase in EAT volume is associated with a 0.12 mm expansion in LA diameter, independent of body mass index (BMI) [15]. Beyond structural changes, EAT has been linked to LA dysfunction—specifically reduced reservoir and conduit strain—even in asymptomatic individuals [14,15,16,17]. Furthermore, the EAT burden correlates with the incidence of AF [18,19]. Some evidence suggests that EAT accumulation may drive the early inflammatory stage of atrial myopathy [20]. Collectively, these findings suggest that EAT is a distinct, modifiable risk factor for LA remodeling.

The generalizability of these findings to the geriatric population remains uncertain, as the majority of existing studies have focused on middle-aged cohorts. Aging is associated with a distinct cardio-metabolic phenotype, characterized by a redistribution of adipose tissue—specifically, an increase in visceral and ectopic fat concurrent with a decrease in subcutaneous depots [21,22,23]. Simultaneously, the aging heart undergoes intrinsic structural and functional alterations, such as myocardial stiffening and fibrosis driven by cumulative hemodynamic loads [24]. These age-related degenerative changes act as dominant drivers of atrial remodeling, potentially overshadowing the influence of EAT. Furthermore, the “obesity paradox” observed in older cohorts—that higher adipose accumulation is occasionally linked to better prognosis—challenges the conventional view that adipose tissue is uniformly deleterious in advanced age [25,26]. Consequently, it remains unclear whether the relationship established between EAT and LA remodeling persists in older adults. Therefore, discerning the specific impact of EAT on LA size in the older population requires dedicated investigation.

Therefore, this cross-sectional study was conducted to explore the relationship between EAT and LA size in an older population. By elucidating this association, we hope to clarify whether EAT remains a modifiable risk factor in the geriatric population, thereby assisting in the effective management of cardiovascular health.

## 2. Materials and Methods

### 2.1. Study Participants

This study was a retrospective analysis of clinical data collected from an older adult cohort at Peking Union Medical College Hospital. A total of 353 participants aged 65 years and older underwent comprehensive medical examinations, including medical history, physical examinations, biochemical tests, computed tomography, and echocardiography. The exclusion criteria included the presence of severe cardiac diseases, such as myocardial infarction, severe valvular heart disease, cardiomyopathy, atrial fibrillation, heart failure, and previous cardiac surgery. Individuals with these conditions were excluded as they are known to independently alter LA structure or systemic hemodynamics, which could confound the EAT-LA relationship. Patients with active infections or malignant diseases, and those who had experienced major surgery or trauma within the previous 3 months were also excluded.

### 2.2. Clinical Data Collection

All clinical data were collected from medical records, physical examinations, and biochemical tests. Demographic parameters included age, gender, and body mass index (BMI). Blood pressure measurements encompassed systolic blood pressure (SBP) and diastolic blood pressure (DBP). History of hypertension, diabetes, or fatty liver disease was recorded. Biochemical tests included total cholesterol (TC), low-density lipoprotein cholesterol (LDL-C), high-density lipoprotein cholesterol (HDL-C), triacylglycerol (TG), apolipoprotein A1 (APOA1), apolipoprotein B (APOB), lipoprotein(a) (LP(a)), free fatty acids (FFAs), glycosylated hemoglobin A1c (HbA1c), C-reactive protein (CRP), and erythrocyte sedimentation rate (ESR).

### 2.3. CT and Epicardial Fat Measurements

All patients underwent CT scans performed with a dual-source computed tomography scanning system (Somatom Definition Flash, Siemens, Forchheim, Germany) without contrast enhancement. The CT images were imported to a dedicated workstation (Syngo with Volume application, Siemens, Forchheim, Germany) for the measurement of EAT. EAT was defined as adipose tissue located within the visceral pericardium [16,27]. The pericardial contours were manually traced from the pulmonary artery bifurcation to the diaphragm. EAT was then quantified semi-automatically as the sum of all voxels within the range of −190 to −30 HU in the traced region (Figure S1) [28,29]. EAT volume was measured in cubic centimeters (cm^3^). Trained radiologists were blinded to the participants’ clinical information.

### 2.4. Echocardiographic Measurements

Echocardiographic measurements were obtained by experienced sonographers using Vivid E9 color Doppler ultrasound systems (GE HealthCare, Horten, Norway), following the guidelines of the American Society of Echocardiography. LA diameter was measured at end-systole using the parasternal long-axis view via echocardiography. Left atrial enlargement was defined as diameter greater than 39 mm in males, and greater than 38 mm in females.

### 2.5. Statistical Analysis

Continuous data were presented as means ± standard deviations or medians with interquartile ranges, while categorical data were expressed as numbers and percentages. Normality was assessed using the Kolmogorov–Smirnov test. Group comparisons of continuous variables were conducted using independent-samples *t*-tests for normally distributed variables and Mann–Whitney U tests for non-normally distributed variables. Fisher’s exact tests and chi-squared tests were used to compare categorical variables.

The association between EAT and LA diameter was initially studied by simple linear regression. Univariate linear regression analyses were performed to identify potential predictors of LA diameter. Variables with statistical significance (*p* < 0.05) in univariate analyses that remained after collinearity adjustment using variance inflation factors (VIF; threshold < 5) were selected for stepwise multivariable modeling. Model fit was assessed using R^2^, F-statistic, and *p* values. Bootstrap resampling (1000 replications) was used to confirm the stability of the estimates.

A generalized additive model (GAM) provided a flexible approach to exploring the shape of the relationship, applying smoothing functions without assuming a predefined relationship [30]. The GAM was fitted with a two-dimensional smooth term for age, EAT, and LA diameter. The effective degrees of freedom (EDFs) and deviance explained were calculated. Based on the World Health Organization (WHO)’s observation that health status and intrinsic capacity decline rapidly after age 75, the participants were stratified into two groups by age: 65–74 years (young-old) and ≥75 years (old-old) [31]. Subgroup analyses were conducted to investigate whether the effect of EAT-LA differed across age groups, and interaction effects were tested.

Restricted cubic spline (RCS) was employed to investigate the potential nonlinear association between EAT and LA diameter, adjusting for age, gender, and body mass index (BMI). Nonlinearity was assessed using analysis of variance (ANOVA). The threshold for EAT was mathematically defined as the point on the fitted RCS curve where the absolute rate of change in the slope (approximating the second derivative) was maximized. The 95% confidence interval (CI) for this threshold was derived via 500 bootstrap resamples. Subsequently, piecewise linear regression was conducted to determine the regression coefficients and significance levels for the segments before and after the threshold.

Statistical analyses were conducted using SPSS (version 26.0) and R (version 4.4.0), with packages including ggforestplot (version 0.1.0) for forest mapping, mgcv (version 1.9.3) for the GAM, rms (version 8.0.0) for RCS, and ggplot2 (version 4.0.1) for visualization. *p* < 0.05 was considered statistically significant.

## 3. Results

### 3.1. Clinical Characteristics of the Study Population

**Figure 1:**
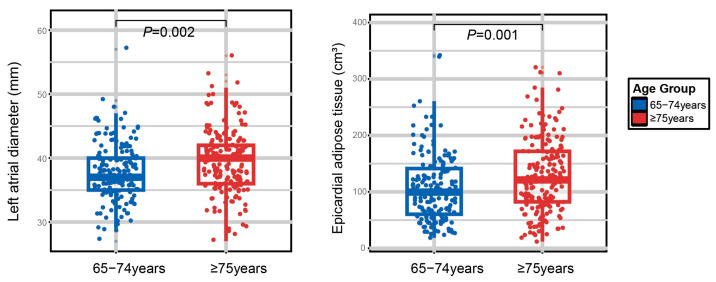
Comparison of left atrial (LA) diameter and epicardial adipose tissue across age groups. Boxplots illustrate the distribution of LA diameter and EAT in patients aged 65–74 years old versus those aged ≥ 75 years old. The horizontal line within each box represents the median, the box boundaries represent the 25th and 75th percentiles (interquartile range, IQR), and the whiskers extend to the most extreme data points within 1.5 times the IQR. *p* values were calculated using independent-samples *t*-test and Mann–Whitney U test and indicate a significant increase in both LA diameter and EAT in the older age group.

Baseline characteristics are summarized in Table 1. This study enrolled 353 participants aged 65–101 years old, with a median age of 75 years. The WHO describes an accelerated decline in health status and intrinsic capacity after approximately 75 years of age. Accordingly, the participants were stratified into two groups by age: 65–74 years (young-old) and ≥75 years (old-old) [31]. Most participants were male (87.3%). As expected, the old-old group exhibited a higher prevalence of hypertension and diabetes, poorer blood pressure and glycemic control, and a greater systemic inflammatory burden. They also had greater EAT and larger LA diameters than the young-old group (Figure 1).

### 3.2. Linear Regression Analysis of Epicardial Adipose Tissue and Left Atrial Diameter

**Figure 2:**
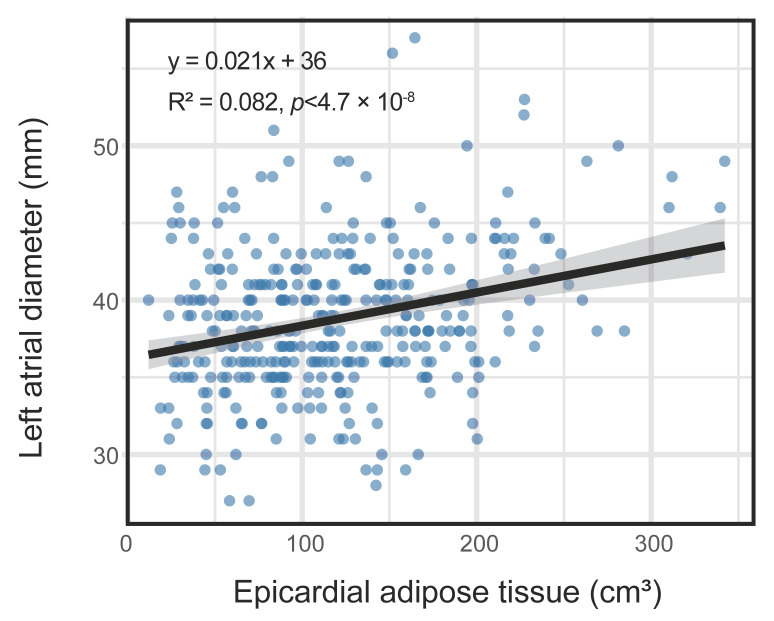
Linear association between epicardial adipose tissue (EAT) and left atrial (LA) diameter. The scatter plot illustrates the relationship between EAT and LA diameter. Each point represents a patient. The solid black line indicates the best-fit line from simple linear regression analysis. The shaded gray area represents the 95% confidence interval.

We first explored the association between EAT and LA diameter with a linear regression model. Simple linear regression analysis revealed a statistically significant but weak positive correlation (R^2^ = 0.082, *p* < 0.001, Figure 2), which accounted for 8.2% of the variance in LA diameter. This result suggested that EAT is positively associated with LA diameter, though there may be other factors with a significant influence on LA diameter. The results of the univariate and multivariate linear regression analyses are escribed below.

#### 3.2.1. Univariate Analysis

Prior to multivariable modeling, univariate linear regression was performed to identify potential predictors of LA diameter. The univariate results (Table 2) showed significant positive associations with LA diameter for age, gender, BMI, hypertension, EAT, and HbA1c. Negative associations were observed for LDL-C, HDL-C, TC, APOA1, and APOB. No significant associations were found for diabetes, TG, LPA, FFA, CRP, or ESR.

#### 3.2.2. Multivariable Linear Regression

**Figure 3:**
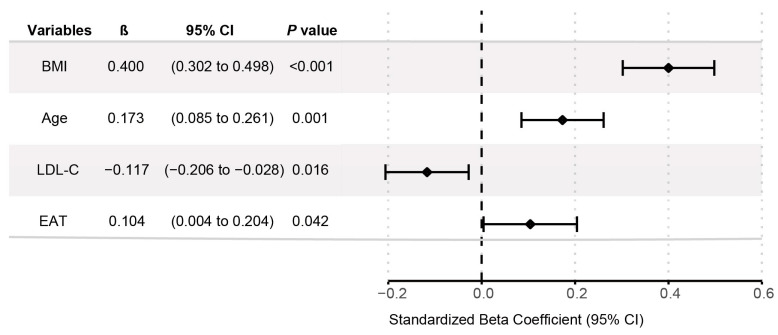
Forest plot of multivariable linear regression analysis predictors of left atrial diameter. The plot presents standardized beta coefficients, 95% confidence intervals, and *p* values for each variable. Abbreviations: β, standardized beta coefficient; CI, confidence interval; BMI, body mass index; LDL-C, low-density lipoprotein cholesterol.

A stepwise multivariable linear regression was performed to identify independent predictors of LA diameter. The process began with variables significant in univariate analysis (*p* < 0.05) after collinearity adjustment (variance inflation factors [VIF] < 5 for all included variables, indicating low multicollinearity). The final model (Table 3) retained BMI, age, LDL-C, and EAT as significant predictors. Age was positively associated with LA diameter (B = 0.092, *p* < 0.001), with an approximate 0.9 mm increase per decade of age. Similarly, EAT showed a positive association (B = 0.008, *p* = 0.042), indicating that for every 100 cm^3^ increase in EAT volume, LA diameter increases by approximately 0.8 mm. In addition, BMI (B = 0.681, *p* < 0.001) was positively associated with LA diameter, while LDL-C showed a negative association (B = −0.665, *p* = 0.013). The model was statistically significant (R^2^ = 0.245, F = 29.544, *p* < 0.001), explaining 24.5% of the LA diameter variation. Bootstrap estimates (1000 replications) confirmed the stability of the findings (Figure 3). These results suggest that EAT is an independent predictor of LA diameter, even after adjusting for age, BMI, and LDL-C.

### 3.3. Interaction Between Age and Epicardial Adipose Tissue on Left Atrial Diameter

To investigate the overall effect of age and EAT on left atrial remodeling, we fitted a generalized additive model (GAM) with two-dimensional smooth terms for age and EAT. A contour map is presented in Figure S2, with color gradients representing predicted LA diameter values with age (x-axis) and EAT (y-axis). The predicted LA diameter increased with advancing age and higher EAT. Those with the maximum predicted LA diameter were at an advanced age and had the highest EAT level.

Next, a subgroup analysis was conducted by dividing the participants into two age groups (Figure S3). The effective degree of freedom (EDF) of the smooth term for EAT in individuals aged over 75 years and older was 2.26 (*p* < 0.001), accounting for 9.9% of the variance. In contrast, in the younger group, the EDF was 1.70 (*p* = 0.002), explaining 7.9% of the variance. An interaction test between the age groups showed no statistically significant result (*p* = 0.967). These results suggest that EAT is an independent predictor of left atrial enlargement, and this association does not change among different age groups.

### 3.4. Nonlinear Association Between Epicardial Adipose Tissue and Left Atrial Diameter

**Figure 4:**
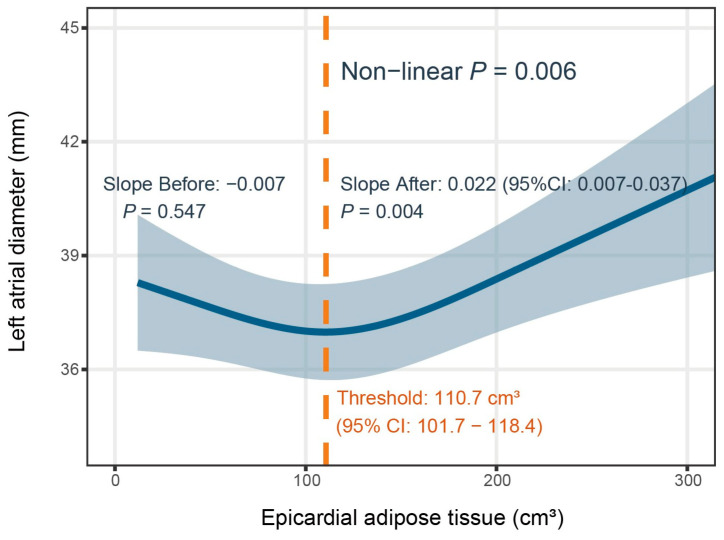
Non-linear association between epicardial adipose tissue and left atrial diameter. The blue solid line shows the J shaped association, and the light blue area represents the 95% confidence interval. A dashed line marks the threshold, with annotations for the regression slopes and *p* values.

Due to the poor explanatory performance of the linear model (Figure 2), we proposed a nonlinear relationship between the two variables. This hypothesis was confirmed using a restricted cubic spline (RCS) model. As illustrated in Figure 4, after adjusting for age, gender, and BMI, the model revealed a J-shaped curve, with an inflection point at 110.7 cm^3^ (95% CI: 101.7–118.4). Piecewise linear regression was used to assess the significance of the association before and after the threshold. ANOVA was used to test nonlinearity. Before the threshold, the curve had a slightly downward slope with no significant association (*p* = 0.547). Beyond the threshold, the slope increased significantly, indicating a more rapid expansion of the left atrium. Specifically, when EAT exceeded 110.7 cm^3^, the LA diameter increased by 0.22 mm for every 10 cm^3^ increase in EAT (B = 0.22, *p* = 0.004). Overall, the effect of EAT on LA diameter was of nonlinear (*p* for non-linearity = 0.006), suggesting that those with EAT values greater than 110.7 cm^3^ are at a markedly accelerated risk of left atrial remodeling.

## 4. Discussion

This study examined the relationship between EAT volume and LA diameter in an older population. To the best of our knowledge, this is the first study to demonstrate a distinct J-shaped curve in this relationship, differing from the simple linear regression typically reported in the general population. We identified elevated EAT volume as an independent factor associated with LA remodeling, characterized by its specific nonlinearity: an EAT volume exceeding 110.7 cm^3^ was significantly associated with a higher likelihood of left atrial enlargement.

Our findings are consistent with previous studies demonstrating a positive correlation between EAT volume and left atrial dimensions [12,13,14,15,18]. A meta-analysis by Mancio et al. [15], which included 19 studies, highlighted that EAT is independently associated with left atrial dilation (pooled β-coefficient: 0.12 mm). Beyond structural changes, studies in the general population have linked increased EAT to an elevated risk of left atrial dysfunction and atrial fibrillation [4,13,17,18,19,20]. While our study confirms the general positive EAT-LA link across age subgroups, further analysis reveals that the relationship deviates from the linear relationship typically reported in general population. Our study reveals a distinctive J-shaped association specifically in older adults with an identified threshold. When EAT beyond 110.7 cm^3^, the slope of the EAT-LA association increased significantly. Specifically, for every 10 cm^3^ increase in EAT above this level, the LA diameter increased by 0.22 mm. This suggests that the impact of EAT on the geriatric heart is not uniform. This nonlinearity underscores the need to explore the specific pathophysiological thresholds where EAT transitions from benign to pathological.

While the precise mechanisms underlying this threshold pattern remain unknown, it is likely attributable to mechanical and paracrine factors. Under physiological conditions, EAT has a cardio-protective role and acts as a mechanical buffer [1]. However, as emphasized by previous studies, EAT resides within the limited space of pericardial cavity [2,3]. When EAT accumulation exceeds the critical inflection point (e.g., over 110.7 cm^3^), excessive EAT may occupy the limited pericardial cavity space, exerting mechanical compression on the heart and impairing diastolic filling, thereby contributing to atrial dilation.

Beyond mechanical compression, a profound biological shift occurs alongside EAT expansion. EAT may trigger “cardio-lipotoxicity”—an excessive infiltration of lipid mediators (e.g., diacylglycerol, ceramides) into the adjacent atrial myocardium, leading to mitochondrial dysfunction and oxidative stress [8]. Additionally, excessive EAT depots may drive a transition from physiologic adipose tissue to a pro-inflammatory and fibrotic phenotype, where the local secretion of cytokines and adipokines (e.g., leptin, interleukin-1β, interleukin-6, resistin) may become sufficient to induce structural remodeling [10]. The paracrine and vasocrine toxicity may trigger coronary microvascular dysfunction (CMD). As detailed by Lanza et al. [32], CMD drives myocardial ischemia and structural abnormalities, subsequently promoting myocardial stiffening and diastolic dysfunction. Furthermore, it is worth noting that EAT may also be linked to atrial electrophysiological remodeling, contributing to cardiac electrical disorders [33,34]. Thus, the steep rise in LA diameter above the threshold likely reflects the compounding effects of both mechanical compression and inflammatory injury.

This biphasic mechanical and biological behavior offers a plausible explanation for the “obesity paradox” frequently observed in geriatric cardiology, such as the J or U-shaped associations between BMI and mortality [35,36,37,38,39]. The relative flat slope below 110.7 cm^3^ suggests that mild-to-moderate EAT acts as a metabolic reserve, conferring a neutral or potentially protective effect against aging. However, our results indicate that this protection is finite. Once EAT accumulation surpasses the threshold, the dual burden of mechanical compression and paracrine toxicity may overwhelm compensatory mechanisms. This transition from a physiologic to a pathologic state highlights the importance of monitoring excessive EAT accumulation in older adults.

Our findings have practical clinical implications. For older adults, employing a threshold of EAT > 110.7 cm^3^ may improve risk stratification, enabling targeted precision screening to identify early LA changes before symptoms appear. EAT assessment could complement conventional parameters (e.g., BMI, lipid profiles) to provide a more comprehensive cardiovascular risk evaluation. Patients with EAT volumes exceeding this threshold, particularly those of advanced age, may benefit from monitoring and lifestyle interventions—such as appropriate exercise prescriptions or pharmacological management—to attenuate these underlying risk factors [2]. In this context, SGLT2 inhibitors represent a promising therapeutic approach [2]. Emerging evidence confirms that SGLT2 inhibitors exert anti-arrhythmic effects and significantly reduce the risk of atrial fibrillation [40]. These benefits appear to be mediated, at least in part, by targeting the metabolic and anti-inflammatory pathways within adipose tissue. Mechanistic insights suggest that SGLT2 inhibition promotes a metabolic shift towards fatty acid oxidation and ketone body production (e.g., β-hydroxybutyrate), a process linked to adiponectin upregulation and the suppression of pro-inflammatory cytokines in adipose tissue, including EAT [41]. By ameliorating the inflammatory microenvironment of EAT, SGLT2 inhibitors may mitigate downstream myocardial injury and stabilize atrial electrophysiology.

Several limitations of this study should be acknowledged. First, the cross-sectional design precludes definitive causal inference. The prognostic value of the EAT threshold identified for incident cardiovascular events requires validation in longitudinal cohorts. Second, the single-center setting, modest sample size, and male predominance may limit the generalizability of our findings. Third, due to the retrospective method of data collection, we relied on LA diameter rather than the LA volume index (LAVI), a more sensitive biomarker, without comprehensive assessment of left ventricular diastolic function (e.g., E/e’). This restricted our ability to fully characterize atrial geometry and elucidate the mechanistic link between EAT and diastolic dysfunction. Finally, despite adjusting for multiple covariates, the possibility of residual confounding from unmeasured factors remains (e.g., genetics factors, medication regimens).

## 5. Conclusions

In conclusion, this study demonstrates an independent association between elevated EAT and LA diameter in older adults, characterized by a J-shaped curve with an inflection point at 110.7 cm^3^. These findings provide insights into the relationship between EAT and cardiac structural alterations, suggesting EAT as a promising biomarker for atrial remodeling. Monitoring EAT volume, particularly when it exceeds this threshold, may provide additional value for cardiovascular risk stratification in older adults. Longitudinal studies are needed to validate causal links and determine whether targeting EAT can attenuate cardiovascular morbidity.

## Figures and Tables

**Table 1:** Baseline Characteristics of the clinical data.

Variables	All N = 353	Age 65–74 Years Old N = 167	Age ≥ 75 Years Old N = 186	*p* Value
Age, years	75 (68, 83)	68 (66, 71)	82 (79, 88)	-
Male	308 (87.3)	139 (83.2)	169 (90.9)	0.038
BMI, kg/m^2^	25.28 ± 2.79	25.30 ± 2.33	25.26 ± 3.15	0.880
SBP, mmHg	132 ± 17	127 ± 15	137 ± 17	<0.001
DBP, mmHg	72 ± 9	75 ± 8	70 ± 10	<0.001
Hypertension	248 (70.3)	101 (60.5)	147 (79.0)	<0.001
Diabetes	121 (34.3)	46 (27.5)	75 (40.3)	0.013
Fatty liver disease	136 (38.6)	62 (37.1)	74 (40.0)	0.586
TC, mmol/L	4.16 ± 0.92	4.24 ± 0.94	4.09 ± 0.90	0.116
LDL-C, mmol/L	2.61 ± 0.84	2.68 ± 0.83	2.55 ± 0.85	0.127
HDL-C, mmol/L	1.34 ± 0.33	1.35 ± 0.33	1.33 ± 0.33	0.572
TG, mmol/L	1.23 (0.94, 1.77)	1.26 (0.94, 1.73)	1.22 (0.91, 1.82)	0.588
APOA1, g/L	1.45 ± 0.23	1.47 ± 0.23	1.42 ± 0.23	0.065
APOB, g/L	0.87 ± 0.23	0.89 ± 0.21	0.85 ± 0.24	0.091
LP(a), mg/L	83 (33, 214)	85 (34, 232)	80 (31, 202)	0.644
FFA, μmol/L	428 (327, 562)	399 (298, 517)	466 (345, 601)	<0.001
HbA1c, %	6.13 ± 0.99	5.95 ± 0.65	6.30 ± 1.19	0.001
CRP, mg/L	0.68 (0.35, 1.51)	0.57 (0.27, 1.12)	0.82 (0.40, 1.81)	0.003
ESR, mm/h	8 (4, 13)	5 (2, 9)	8 (6, 16)	<0.001
EAT, cm^3^	111.07 (69.55, 158.92)	99.51 (60.10, 142.59)	121.17 (82.10, 171.93)	0.001
LAD, mm	38.73 ± 4.76	37.89 ± 4.49	39.49 ± 4.88	0.002

Quantitative data are presented as means ± standard deviations, or as medians with interquartile ranges. Qualitative data are presented as number (%). Abbreviations: BMI, body mass index; SBP, systolic blood pressure; DBP, diastolic blood pressure; TC, total cholesterol; LDL-C, low-density lipoprotein cholesterol; HDL-C, high-density lipoprotein cholesterol; TG, triacylglycerol; APOA1, apolipoprotein A1; APOB, apolipoprotein B; LP(a), lipoprotein(a); FFA, free fatty acids; HbA1c, glycosylated hemoglobin A1c; CRP, C-reactive protein; ESR, erythrocyte sedimentation rate. EAT, epicardial adipose tissue; LA diameter, left atrial diameter.

**Table 2:** Univariate linear regression analysis of predictors for left atrial diameter.

Variables	Univariable Linear Regression	
	β (95% CI)	*p* Value
Age	0.164 (0.060, 0.267)	0.002
Gender	0.157 (0.054, 0.261)	0.003
BMI	0.435 (0.341, 0.530)	<0.001
Hypertension	0.210 (0.107, 0.313)	<0.001
Diabetes	0.044 (−0.061, 0.149)	0.830
Fatty liver disease	−0.097 (−0.201, 0.008)	0.070
EAT	0.286 (0.185, 0.386)	<0.001
LDL-C	−0.184 (−0.287, −0.081)	0.001
HDL-C	−0.154 (−0.258, −0.051)	0.004
TC	−0.204 (−0.307, −0.102)	<0.001
TG	0.053 (−0.052, 0.157)	0.325
APOA1	−0.173 (−0.278, −0.070)	0.001
APOB	−0.158 (−0.262, −0.054)	0.003
LPA	0.026 (−0.080, 0.131)	0.633
FFA	0.019 (−0.086, 0.124)	0.726
CRP	0.057 (−0.048, 0.163)	0.285
ESR	0.023 (−0.082, 0.128)	0.663
HbA1c	0.120 (0.016, 0.224)	0.024

Abbreviations: β, standardized regression coefficient; CI, confidence interval; BMI, body mass index; EAT, epicardial adipose tissue; LDL-C, low-density lipoprotein cholesterol; HDL-C, high-density lipoprotein cholesterol; TC, total cholesterol; TG, triglycerides; APOA1, apolipoprotein A1; APOB, apolipoprotein B; LPA, lipoprotein(a); FFA, free fatty acids; CRP, C-reactive protein; ESR, erythrocyte sedimentation rate; HbA1c, glycated hemoglobin A1c.

**Table 3:** Multivariable linear regression analysis for left atrial diameter.

Variables	Left Atrial Diameter					
	B	SE	β	t	*p* Value	VIF
BMI	0.681	0.087	0.400	7.822	<0.001	1.216
Age	0.092	0.026	0.173	3.614	0.001	1.062
LDL-C	−0.665	0.266	−0.117	−2.499	0.013	1.029
EAT	0.008	0.004	0.104	2.040	0.042	1.213
R^2^	0.245					
F	29.544 ***					

*** *p* < 0.001. Abbreviations: B, unstandardized coefficient; SE, standard error; β, standardized coefficient; t, t-statistic; VIF, variance inflation factor; BMI, body mass index; LDL-C, low-density lipoprotein cholesterol; EAT, epicardial adipose tissue.

## Data Availability

The original contributions presented in this study are included in the article/Supplementary Materials. Further inquiries can be directed to the corresponding authors.

## Supplementary Materials

The following supporting information can be downloaded at: https://www.mdpi.com/article/10.3390/jcdd13020078/s1, Figure S1. Schematic diagram of epicardial adipose tissue (EAT). Figure S2. Contour plot of left atrial diameter across age and epicardial adipose tissue; Figure S3. Predicted left atrial diameter in relation to epicardial adipose tissue, stratified by age group.

## Abbreviations

The following abbreviations are used in this manuscript:

EAT	epicardial adipose tissue
LA	left atrial
BMI	body mass index
SBP	systolic blood pressure
DBP	diastolic blood pressure
TC	total cholesterol
LDL-C	low-density lipoprotein cholesterol
HDL-C	high-density lipoprotein cholesterol
TG	triacylglycerol
APOA1	apolipoprotein A1
APOB	apolipoprotein B
LP(a)	lipoprotein(a)
FFA	free fatty acids
HbA1c	glycosylated hemoglobin A1c
CRP	C-reactive protein
ESR	erythrocyte sedimentation rate
VIF	variance inflation factors
GAM	Generalized additive model
RCS	Restricted cubic spline
WHO	World Health Organization
