# Supplementary material for: Epicardial Adipose Tissue Volume and Left Atrial Remodeling: A J-Shaped Association in Older Adults
Source: J Cardiovasc Dev Dis. 2026 Feb 4;13(2):78. doi: 10.3390/jcdd13020078 (PMC12942443; doi:10.3390/jcdd13020078)
Supplement: Supplementary file 1 [file jcdd-13-00078-s001.zip › jcdd-4091862-supplementary.pdf]

Figure S1

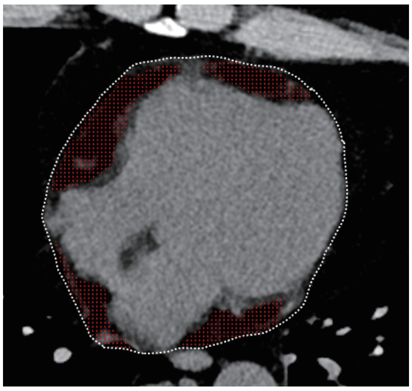

Figure S1. Schematic diagram of epicardial adipose tissue (EAT).
The white dashed line delineates the pericardium, while the blue-dotted area represents EAT.

Figure S2

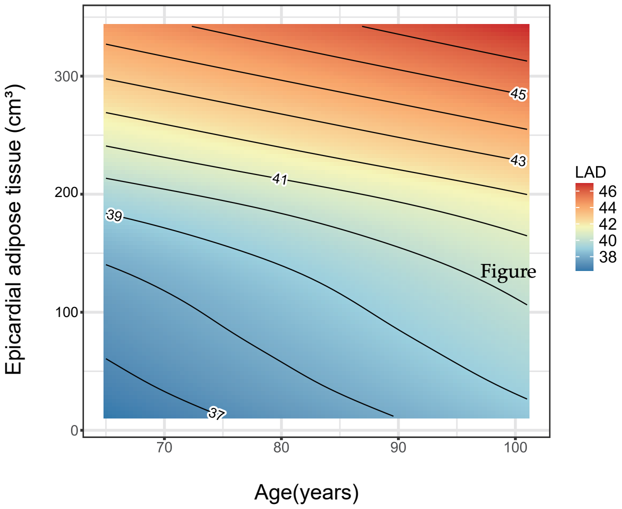

Figure S2. Contour plot of left atrial diameter across age and epicardial adipose tissue.
Based on the generalized additive model (GAM), the overall effect of age and EAT on LA diameter was illustrated. The color gradients indicate predicted LA diameter. The black lines represent LA diameter contour lines, with corresponding numbers labelled on them.

Figure S3

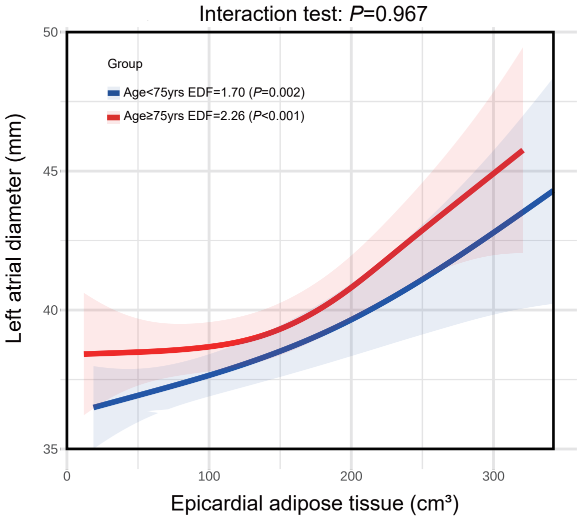

Figure S3. Predicted left atrial diameter in relation to epicardial adipose tissue, stratified by age group.
Blue and red represent the young-old (65-74years) and old-old (≥75years) group respectively.
Abbreviations: EDF, effective degrees of freedom.
